# Conducting Virtual Focus Groups During the COVID-19 Epidemic Utilizing Videoconferencing Technology: A Feasibility Study

**DOI:** 10.7759/cureus.23540

**Published:** 2022-03-27

**Authors:** Ghadah Almujlli, Rola Alrabah, Abdulmajeed Al-Ghosen, Fadi Munshi

**Affiliations:** 1 Simulation and Skills Development Center, Princess Nourah Bint Abdulrahman University, Riyadh, SAU; 2 Simulation and Medical Education, Princess Nourah Bint Abdulrahman University, Riyadh, SAU; 3 Emergency Medicine, King Abduallah bin Abdulaziz University Hospital, Riyadh, SAU; 4 Assessment, Saudi Commotion for Health Specialties, Riyadh, SAU

**Keywords:** qualitative research, feasibility, virtual environment, qualitative evaluation, focus group

## Abstract

Background

Due to the coronavirus disease 2019 (COVID-19) pandemic, the world has seen a surge in utilizing videoconferencing technology. It can be a useful approach for qualitative research. This study describes the feasibility of virtual focus groups in qualitative research.

Methods

Videoconferencing software was used to conduct virtual focus groups. A dry run was conducted a day before the focus group to ensure the research team was acquainted with the software on the focus group day.

Results

Using distance videoconferencing software was cost-effective compared to face-to-face focus groups. The moderator was responsible for leading the discussion virtually. Unlike in-person focus groups, the virtual focus group scheduling was flexible, and it was easier to find replacements for participants who dropped out.

Conclusion

This study found that conducting virtual focus groups utilizing videoconferencing software was time-saving and cost-efficient compared to face-to-face focus groups.

## Introduction

Coronavirus disease 2019 (COVID-19) caused several responses from governmental institutions and enforced lockdown on all educational institutions in Saudi Arabia for the second semester of 2020. The lockdown led institutions to complete the academic year virtually [1]. During this lockdown period, a quarantine was enforced that has limited the ability to conduct in-person research activities that are not clinically oriented.

Due to the worldwide lockdown and subsequent quarantine, the world has seen a surge in videoconferencing technology to conduct virtual meetings and continue work during the quarantine period [2-6]. While this technology helps facilitate distance work, it can be a useful approach for qualitative research and, specifically, focus groups [7-9]. Due to quarantine and COVID-19 precautions, it was not feasible to utilize face-to-face focus groups as a qualitative research methodology. Using videoconferencing technology to conduct virtual focus groups was a more efficient approach that ensured compliance with COVID-19 precautions enforced by the government.

A focus group is defined as a group of individuals selected by researchers to discuss and comment on a research element based on personal perceptions and experience [10]. While a face-to-face focus group is traditionally conducted in a research facility equipped with audio and visual recording systems, a virtual focus group is conducted in real-time utilizing a videoconferencing program [8,11]. A few studies were described using this methodology, and many concluded that the quality of data obtained from virtual focus groups is similar to the face-to-face focus groups.

For example, in Williams et al. study, they explored the public’s experience of social distancing in the current COVID-19 pandemic using videoconferencing technology. The study reported that utilizing a videoconferencing application (Zoom™) has enabled the possibility to meet and conduct focus groups during social isolation [12]. Another study conducted in Canada by Gray et al. has shown that utilizing videoconferencing software has the benefit of accessibility of participants and is cost-effective when compared to traditional face-to-face focus groups [13].

Qualitative research that utilized distance real-time approach in focus groups or interviews and the reported benefits and drawbacks of using videoconferencing software is described in a table in this article, which aimed to identify the best method for virtual focus groups and clarify the benefits and drawbacks of the virtual focus groups. It has been developed by searching for qualitative studies that utilized virtual focus groups in the study methodology. First, studies that conducted focus groups using an online platform with no videoconferencing application were excluded. Next, a member of the research team reviewed the studies. They summarized the studies’ aim, study design, study methods, reported findings, reported benefits of virtual focus groups, and noted virtual focus group approach drawbacks. Finally, after excluding the studies that did not fit the inclusion criteria, the studies were reviewed and summarized.

The most reported benefits of conducting a virtual focus group were flexibility in scheduling, recruiting participants from rural or hard-to-reach areas, and being more cost-effective than a face-to-face focus group [9,13]. However, the drawback reported pointed out that using this approach might result in technical issues during the focus group that might lead to recording issues [14]. Also, the nature of the method might lead to omitting some subjects without access to a private network. Finally, the major drawback reported in the literature is that detecting non-verbal cues is difficult [13].

Feasibility factors found in the literature were required technical support, videoconferencing software operation, ethical approval and participant’s consent, and cost of conducting virtual focus groups. Studies found that focus groups can be performed virtually if they were carefully planned, and several factors related to feasibility have been considered.

While the literature showed that it is feasible to implement virtual focus groups, to the authors’ knowledge, no published studies utilized virtual focus groups in Saudi Arabia [11-13]. Therefore, this study aimed to investigate the feasibility of conducting focus groups using videoconferencing technology in Saudi Arabia under the conditions of pandemic lockdown.

## Materials and methods

This study was a qualitative study that utilized the grounded theory approach. The data collection method for the study was focus groups. The focus group aimed to investigate experts’ opinions on the quality indicators of healthcare simulation scenarios. Initially, the first focus groups were to be in person. They were scheduled to be conducted in June and July 2020 at the simulation and skills development center in Princess Nourah Bint Abdul Rahman University, Riyadh, Saudi Arabia. The planned focus groups were part of a research project investigating the indicators that determined the quality level of healthcare simulation scenarios. However, after the enforced quarantine caused by COVID-19, the face-to-face focus group approach was not possible, and it was mandatory to shift the approach to a virtual focus group.

After reviewing the studies that focused on conducting virtual focus groups and exploring the features of each platform that was referenced in the studies, Zoom™ was chosen for conducting the focus groups [12,13,15,16]. The software has been recently utilized in qualitative research [8,13,15]. The application is convenient to use due to the specific features that it offers to its users. It can be used on any electronic device (laptop, personal desktop, tablet, etc.); meetings can be secured with encryption; it allows the host and attendees to share files, and several other features make it an appropriate application to achieve the purpose of the study [16].

The shift from a traditional focus group to a virtual one required several changes in the study protocol. The format of a virtual focus group has been reported to limit the interactions observed in the focus groups due to the absence of a shared physical setting [11,13,17]. Consequently, several considerations were taken to ensure that the participants could interact together, similar to the traditional focus group [11]. The studies conducted by Tolhurst et al. and Flynn et al. have developed a protocol for conducting virtual focus groups [9,18]. Both studies were utilized to modify the face-to-face focus group approach to the virtual focus group approach. The estimated costs of conducting the focus groups were based on the research team's estimation of expenses. Table 1 shows the changes implemented in the research protocol.

**Table 1:** Modifications made to shift the focus groups from face-to-face approach to distance approach

Modification	Face-to-face focus group	Virtual focus group
Setting	Large private meeting room to ensure confidentiality.	Research team setting:
	Notebook/computer.	A small quiet room to ensure confidentiality and limit distractions. Computer/laptop with a camera. Recorder. Focus group script.
	Recorder.	Videoconferencing software (Zoom^™^) that has the following features:
	Flip chart paper.	Videoconferencing software (Zoom^™^) that has the following features: Provides high-definition (HD) video and audio. Records focus groups locally or to the software’s cloud. Supports scheduling or starting meetings from different calendar applications (e.g., Outlook, Gmail, ... etc.). Provides the option to chat with participants. Enables file-sharing with participants. Secures focus groups meeting with encryption. Enables the option of requiring the moderator to be present before the focus group meeting starts. Enables and disables a participant or all participants to record the focus group. Uses a passcode to protect a focus group meeting.
	Focus group list of participants.	Participants setting:
	Focus group script.	A small quiet room to ensure confidentiality and limit distractions. A laptop/personal computer/cellphone’s camera must be on to observe the body language.
	Participants' name tags.	
	Watch or clock to track time.	
Number of participants	5-8 participants	5-7 participants
Required technical support	Microphones.	Videoconferencing software.
	Audio system support for the microphones.	Troubleshooting guide for technical issues taken from the software’s website.
Moderator role	Conduct the focus group.	Conduct the focus group.
	Facilitate the discussion.	Facilitate the discussion.
	Operate recording equipment.	Manage the videoconferencing software chat room and meeting.
		Manage the security of the meeting.
Facilitator/assistant moderator role	Observe non-verbal cues.	Observe non-verbal cues.
	Ask questions when invited.	Ask questions when invited.
	Take notes throughout the focus group.	Take notes throughout the focus group.
	Give an oral summary.	Give an oral summary.
	Debrief with the moderator.	Debrief with the moderator.
		Assist with troubleshooting of the software and recording equipment.
Ethical approval and participant’s consent	The consent form is handed in on the focus group day, and the moderator will thoroughly explain the study’s aim and the participant's right to confidentiality and anonymity.	The consent form is sent after receiving the initial approval of the participants.
	The participants will be voice recorded for research purposes.	The participants electronically sign the consent form.
	The participants will review the consent form and are free to ask any questions before signing the consent form.	The consent form is shared with the participants on the day of the focus group.
	A copy of the consent form is offered to each participant.	The moderator will thoroughly explain the study’s aim and the participant's right to confidentiality and anonymity.
		The moderator will inform the participants that the meeting is video recorded.
		The moderator will receive verbal consent from all the participants before starting the focus group.
Estimated costs	Participants’ travel expenses: 200–400 SR, per participant = 3750–6000 SR.	Videoconferencing software license: 60–120 SR/month.
	Technological equipment including microphones and recorders: 1000–1500 SR. Catering: 500–1000 SR. Stationary: 50– 00 SR. Total = 5300–8600 SR.	Recording devices: 400–800 SR. Total = 460–920 SR.

Feasibility factors of virtual focus groups

**Figure 1:**
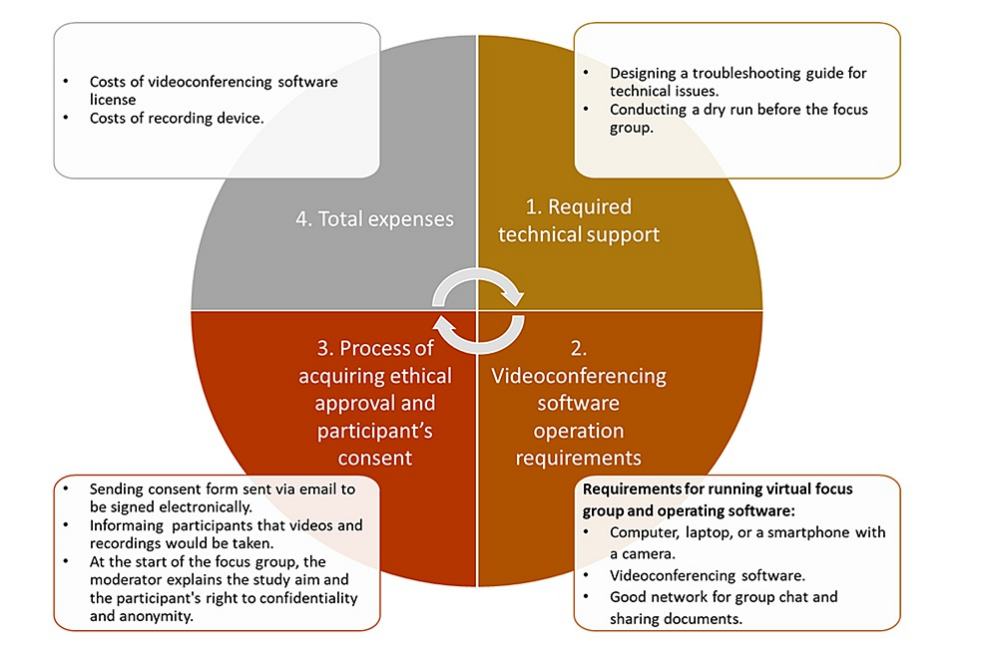
Feasibility factors considered when conducting virtual focus groups

After shifting from face-to-face to virtual focus groups, the research team determined four feasibility factors for virtual focus groups based on the literature review of available literature (Table 1). The feasibility factors involved in virtual focus group protocol required technical support, videoconferencing software operation requirements, the process of ethical approval and participant’s consent, and total expenses. Figure 1 shows the feasibility factors considered when drafting the virtual focus group protocol.

To assess the feasibility factor of virtual focus groups, the research team met after completing the focus group and compared the procedures and processes detailed in the protocol that was designed based on the available literature and previous studies (Table 2), with the outcome of virtual study groups. Table 3 shows the process of determining and summarizing the feasibility factors for virtual focus groups.

**Table 2:** Summary of research findings of qualitative research that used distance approach

(Author, Year) Country	Aim	Research design/methods	Research findings	Reported benefits of distance qualitative research approach	Reported drawbacks of distance qualitative research approach
Williams et al., 2020 [12], United Kingdom (UK)	Explore public experience and perception of social distance and isolation due to COVID-19 pandemic.	The study included five focus groups that took place online in real-time via Zoom^™^.	Three main themes were found: Loss of social interaction, income, routine, motivation, meaning, and self-worth. Adherence to COVID-19 guidelines. Uncertainty of the future.	Enable to meet and conduct focus groups in social isolation.	Not reported.
Gray et al., 2020 [13], Canada	Examine specific attributes of videoconference application that contributes to high-quality interviews.	In-depth interviews were conducted with participants, and four questions were asked about the participant’s perception of utilizing videoconferencing applications via Zoom^™^.	Participants reported a positive outlook on using online focus groups. In addition, the following themes were found: Convenience. Ease of use. Due to familiarity with the environment, it was easier to discuss personal topics. Utilizing different technical devices. Time-saving.	Accessibility to participants. Cost-effectiveness compared to traditional face-to-face interviews. Ease in establishing a connection with the interviewer. Secure data storage. Personal safety. Allows the interviewer to observe non-verbal communication.	Extra charges are required when utilizing videoconferencing software. Technical difficulties. A small percentage of the population might not have access to a private internet connection. Lack of shared physical space resulted in an inability to observe body language and emotional cues.
Akyıldız 2020 [15], Turkey	Examine university students’ perceptions of distance education.	Virtual focus groups were conducted with 12 undergraduate students via Skype^™^.	Students had been negatively affected by the pandemic, feeling despair, anxiety, and boredom. In addition, the change in the education process has led to students feeling isolated, and the lack of interaction with the instructors led to bad habits of time management. The students stated some advantages of distance learning, such as flexibility and taking responsibility for learning.	1. It is practical to conduct online focus groups due to the pandemic. 2. Convenient to researchers due to their familiarity with the online platform. The familiarity was due to the transition from face-to-face discussion to online because of the pandemic.	No drawbacks were reported.
Kite and Phongsavan, 2017 [11], Australia	Provide critical reflection about the utilization of web-based conferencing services to conduct focus groups.	Three face-to-face focus groups and two online focus groups were done using Blackboard Collaborate™. The reflective practice of both approaches was done to decide the differences and similarities.	The data obtained from both approaches were similar. However, technical difficulties were observed between the participants when using the online software. Additionally, there were issues when managing the quality of recordings.	Closely mirrors face-to-face focus group. The observed dynamic in the virtual focus group is similar to the face-to-face focus groups. Online focus group participants provided more insight than the face-to-face focus groups.	Personal technical issues, such as children, background noise, and phone distractions, led to interruption. Compared to face-to-face focus groups, communication was slower, and sometimes the discussion would deviate from the research question. Some participants faced difficulty with hearing each other. The echo resulting from the technical issues caused difficulty in transcribing the focus group. Moderator could not detect non-verbal cues.
Flynn et al., 2018 [9], Canada	Propose two alternative approaches to focus groups that alleviate challenges and barriers faced in qualitative research.	The research used two approaches: The approach was to extend the period of quantitative data collection to facilitate building relationships before qualitative focus groups. Use of videoconference to conduct focus groups.	The approaches resulted in high participation rates (n = 52), rich qualitative data, and cost savings. In addition, both methods were effective when conducting qualitative research in geographically dispersed areas, rural and remote research, and busy clinical environments.	Conducting research in remote sites that are usually left out in qualitative research added a new perspective to the study. It ensured sufficient participation that justified expenses spent to reach remote locations and rural areas. Technology utilized in videoconferencing has enabled overcoming recruitment, distance, and cost challenges when including participants from busy rural clinical environments. Flexibility in scheduling.	Technical issues include but are not limited to video disconnection and poor audio.

**Table 3:** Process of determining the feasibility factors for virtual focus groups

Feasibility factors	Process of deciding feasibility factors
1. Required technical support	Observation of focus group conduction.
2. Videoconferencing software operation requirements	Observation of focus group conduction. Research team assessment of operation requirements after completing virtual focus groups.
3. Ethical approval and participant’s consent	Observation of focus group conduction. Research team assessment of the process to acquire approval and participant’s consent after completing virtual focus groups.
4. Total expenses	Comparing pre-determined costs with final expenses.

Developing virtual focus group protocol

A focus group protocol was drafted that details the role of the research team and the outline of the focus group. It was modified after shifting the approach to fit the virtual focus group, and the questions that will be asked to the experts are put in order from general to specific. The focus group questions were created after an in-depth literature review of available evidence that described quality indicators of simulation scenarios in healthcare education [19-21]. The literature review findings were utilized to draft and finalize the focus group questions and outline the discussion topics.

The sampling technique followed to recruit participants in the study was a purposive sampling technique [22]. The subjects were included based on the specified inclusion and exclusion criteria. Participants included in the focus group must be involved in simulation education, whether in managerial or academic positions; they must have experience in simulation education for more than one year and have been actively engaged in healthcare simulation scenario design or curriculum design [23,24]. Participants who are involved exclusively in the operation of simulation activities were excluded from the study. Participants were selected from LinkedIn and by recommendations from local simulation experts based on the inclusion criteria [25].

All subjects meeting the criteria in LinkedIn were added to a database of experts. The inclusion was based only on their job experience and education which are stated on their public page. A total of 60 subjects were recruited and included in the simulation experts’ database. Additional 15 experts were added based on simulation experts’ recommendations that were consulted by the research team. The proposed number for focus groups was three focus groups, with participants ranging from five to six members in each group with an estimated run time of 60-90 minutes.

Participants were communicated via direct messages on LinkedIn or via emails by the study's principal investigator. The message or email detailed the study purpose, participant’s role, and proposed focus group dates. The participants who replied with their interest to participate were sent an email that included the time and date of the focus group link of the Zoom meeting. A consent form was also sent to the participants. They were instructed to read and sign the consent form, and if they have any inquiries regarding the research, they were instructed to contact the principal investigator via phone or email.

Before the focus group date, the research team conducted a dry run demo focus group. The aim was to test the software's technical settings and ensure that the research team was well acquainted with the software’s platform. The moderator and assistant moderator roles were assigned before the focus group date. Moderator has been trained in conducting qualitative research and has observed the process of the focus groups. The assistant moderator has been briefed on his role before the focus group date. A document detailing the roles of the moderator and the assistant moderator was sent along with the focus group script.

On the focus group day, the moderator was the host of the meeting and focus group discussion. Recording of the focus group was done via the application, and participants were informed beforehand. The moderator facilitated the discussions during the focus group, while the assistant moderator tracked the non-verbal cues and took notes. After the focus group concluded, both moderator and assistant met and drafted a summary of the focus group, topics that emerged, and recommended ways to improve focus group conduction in the future. The study was approved by the Princess Nourah University Institutional Review Board (IRB), and the IRB log number was 19-0105.

## Results

Attributes of participants

The research team conducted three focus groups. The first focus group consisted of five simulation experts with one to five years of experience in simulation education. The second focus group had six participants with expertise ranging from two to seven years in simulation education. The last focus group had six participants with four to eight years of working in simulation education. Of the 16 participants in the focus groups, 13 were recruited from Riyadh city, two from Jeddah, and one from the United States. Table 4 shows detailed demographic data of the participants.

**Table 4:** Attributes of focus group participants

Subject’s attributes		n (%) Total = 16
Gender	Female	10 (63%)
	Male	6 (38%)
Simulation experience	1-2 years	2 (13%)
	2-5 years	9 (56%)
	5-7 years	4 (25%)
	>7	1 (6%)
Role in simulation education	Managerial role	6 (38%)
	Educational role	7 (44%)
	Managerial and educational roles	3 (19%)

Interactions between focus group participants and moderator

During the discussions, the participants took 15-20 minutes to shift their conversations from the moderator to each other. It was noted that some of the participants dominated the discussion, and others deviated from answering the questions given by the moderator. On those occasions, the moderator had to keep the participants focused on the topics of the focus group. Less experienced participants required more prompting to engage in the discussions. Some participants tended to drift from the research question to talk about different topics, which prompted the moderator to divert the conversation back on track.

Additionally, the moderator focused on ensuring recording quality and limiting technical issues that might affect the audio. The assistant moderator followed the topics outlined in the protocol and ensured that all questions were answered. He also assisted in fixing technical issues that accrued to allow the moderator to focus on the discussion. However, he faced difficulty detecting non-verbal cues since the camera focuses on the participants' faces, limiting their observations.

Benefits and drawbacks of the virtual focus group

The dry run conducted before the focus group helped in anticipating technical issues and troubleshooting on the spot. The run time for each focus group ranged from 80 to 100 minutes. The cost of conducting the focus group was far less than the estimated budget for running face-to-face focus groups. The face-to-face focus groups required microphones, audio recorders, refreshments, laptops to present the necessary information to the participants, and office supplies such as pens and papers. However, the virtual focus group expenditure was limited to a license to use the application.

One of the first focus group participants had technical difficulties setting his microphone and was quickly helped by the assistant. There were also network connection issues that occurred during the first focus group with a participant. The connection issues led the host of the focus group to stop sharing folders to minimize the load on the internet connection. In addition, the quality of transcription in the second focus group was affected by one of the participants having an audio issue and echo. This issue led to difficulty following the conversation during the short period that the participants faced the audio technical problem. Finally, due to the nature of the virtual focus group approach, it was difficult for the assistant moderator to observe non-verbal cues from the participants, impacting tracking the participants' non-verbal cues.

Feasibility factors of the virtual focus groups

To assess the feasibility of virtual focus groups, the research team met after the virtual focus group and compared the procedures and processes detailed in the protocol with the protocol's implementation and outcome. When considering conducting focus groups, several factors were considered to ensure proper conduction. Factors related to the feasibility included such as required technical support, videoconferencing software operation requirements, the process of ethical approval and participant’s consent, and total expenses. Table 5 describes the feasibility factors that were considered when conducting virtual focus groups.

**Table 5:** Summary of the feasibility factors of the virtual focus groups

Feasibility factors	Description
1. Required technical support	A troubleshooting guide for technical issues was taken from the software’s website. A dry run was conducted before the focus group date to prepare for different technical issues during the virtual focus group day.
2. Videoconferencing software operation requirements	Computer, laptop, or a smartphone with a camera. Videoconferencing software (Zoom^™^) with the following features: Provides HD video and audio. Records focus groups locally on the software’s cloud. Supports scheduling or starting meetings from different calendar applications. Provides an option to chat with participants. Enables sharing files and documents with participants live or sent during meetings. Secures focus group meetings with encryption. Enables the moderator to choose to be present before the focus group meeting starts. Allows the moderator to secure the focus group meetings with a passcode. Good network for group chat and sharing documents (1.0 megabits per second/600 kilobits per second).
3. Ethical approval and participant’s consent	The consent form was sent via email after receiving the initial approval of the participants. The email informed the participants that videos and recordings would be taken for research purposes and kept confidential. It was stated that a video camera should be on during the focus group, so the research team can observe body language and non-verbal cues. The participants electronically signed the consent form. The consent form was shared with the participants on the day of the focus group. The moderator thoroughly explained the study’s aim and the participant's right to confidentiality and anonymity. The moderator informed the participants that the meeting was video recorded. The moderator will receive verbal consent from all the participants before starting the focus group.
4. Total expenses	Videoconferencing software license = 64.65 SR/month. A total of three-month subscriptions for the duration of the virtual focus group conduction = 193.95 SR. Recording device = 515 SR. Total = 708.95 SR.

## Discussion

The study aimed to describe the experience of conducting focus groups utilizing distance videoconferencing software. Three focus groups were conducted with participants ranging from five to six and run time ranging from 80 to 100 minutes. Using videoconferencing software was cost-effective compared to face-to-face focus groups. Another benefit of utilizing this approach was flexibility in scheduling the focus groups as scheduling with busy participants was more flexible than a face-to-face focus group. Furthermore, it was a possible recruited focus group with participants from different areas.

This study demonstrated the various roles that a research team requires to conduct virtual focus groups. The moderator was responsible for leading the discussion virtually. Simultaneously, the assistant’s role was to support the moderator and analyze body language and verbal input or written comments. Unlike in-person focus groups, the virtual focus group scheduling was flexible, and it was easier to find replacements for participants who opted to drop out.

The finding of this study is similar to previous research [9,11,18]. In the Flynn et al. (2018) study, they compared face-to-face and virtual focus groups. The authors reported that while the two approaches differed in conduction, the virtual focus group was more flexible in timing and convenient when conducting research with participants from other geographical areas [9]. Another study compared the two approaches; they faced several technical difficulties while running the virtual focus group and reported that the quality of data taken from both the face-to-face and virtual focus groups was similar [11].

The downside of the virtual focus groups was majorly focused on technical issues that interfere with the discussions and affect the quality of the recordings, which might lead to difficulty in the transcription of focus groups. The second downside is the difficulty in observing the non-verbal cues of participants. Another drawback was the inability to control the participants' environment. Distractions during the focus group might cause the participant to be distracted and not participate in the focus group.

Lesson learners and recommendations

To conduct virtual focus groups, the research team must consider the features required from the platform and software to host the focus groups. The features needed to run focus groups include providing HD video and audio, recording focus groups locally on the software’s cloud, supporting scheduling or starting meetings from different calendar applications, giving the option to chat with participants, enabling file sharing during sessions, securing focus group meetings with encryption, enabling the moderator to choose to be present before the focus group meeting starts, and allowing the moderator to secure the focus group meetings with a passcode.

Second, running a trial or a dry run of the focus group with the research team and anticipating most issues during the focus group meeting. The dry run is crucial as it will save valuable time that might be lost to solve this issue. It will enable the moderator and assistant moderator to manage the technical problems that might happen promptly. The third recommendation concerns the ethics and consent of participants. The researcher must ensure that participants have signed the consent form or verbally agreed on recording that they understand and agree to participate in the focus group. They also must clarify those participants know that they will be recorded and keep their cameras on for the entirety of the focus group. Finally, because virtual focus groups utilize different technology and approach compared to the in-person focus group, it is crucial to ensure that the device used by the participants and the research team has good audio and camera quality; they also need to have a good network connection to avoid lagging and disconnecting during the focus group meeting.

Limitation 

This study has some limitations. The lack of comparison of the traditional approach to focus groups did not help us compare the two methods. Also, the run time of focus groups was longer than conventional focus groups, which can be attributed to the group dynamics observed in these focus groups [26]. One example of the observed focus group dynamic was participants who dominated the discussion and others who spoke less. This dynamic was observed in the Gratton and O’Donnell (2011) study [26]. Like their approach, the moderator was responsible for ensuring that everyone had their chance to contribute to the discussion [26]. More research could be done to explore and understand the group dynamics in virtual focus groups and how the dynamic affects the data quality.

## Conclusions

In this study, virtual focus groups utilizing videoconferencing software were more time-saving and cost-efficient than face-to-face focus groups. Technical difficulties can be limited if the research team is well trained to address issues that might arise during the focus group. Furthermore, the researcher team should address the most common technical problems that might arise during the virtual focus group. Finally, further research could be done to investigate the differences in group dynamics between the two approaches.
